# Influence of Antiplatelet and Anticoagulant Drug Use on Outcomes after Chronic Subdural Hematoma Drainage

**DOI:** 10.1089/neu.2018.6080

**Published:** 2021-04-05

**Authors:** Michael T.C. Poon, Catherine Rea, Angelos G. Kolias, Paul M. Brennan

**Affiliations:** ^1^ Department of Clinical Neuroscience, Western General Hospital, Edinburgh, United Kingdom.; ^2^ Centre for Clinical Brain Sciences, The University of Edinburgh, Edinburgh, United Kingdom.; ^3^ Department of Haematology, Eastbourne District General Hospital, Eastbourne, United Kingdom.; ^4^ Division of Neurosurgery, Department of Clinical Neurosciences, University of Cambridge and Addenbrooke's Hospital, Cambridge, United Kingdom.; ^5^ Surgery Theme, Cambridge Clinical Trials Unit, Cambridge Biomedical Campus, Cambridge, United Kingdom.

**Keywords:** anticoagulant, antiplatelet, chronic subdural hematoma, functional outcome, recurrence

## Abstract

We aim to describe the outcomes after chronic subdural hematoma drainage (CSDH) management in a large cohort of patients on antithrombotic drugs, either antiplatelets or anticoagulants, at presentation and to inform clinical decision making on the timing of surgery and recommencement of these drugs. We used data from a previous UK-based multi-center, prospective cohort study. Outcomes included recurrence within 60 days, functional outcome at discharge, and thromboembolic event during hospital stay. We performed Cox regression on recurrence and multiple logistic regression on functional outcome. There were 817 patients included in the analysis, of which 353 (43.2%) were on an antithrombotic drug at presentation. We observed a gradual reduction in risk of recurrence for patients during the 6 weeks post-CSDH surgery. Neither antiplatelet nor anticoagulant drug use influenced risk of CSDH recurrence (hazard ratio, 0.93; 95% confidence interval [CI], 0.58–1.48; *p* = 0.76) or persistent/worse functional impairment (odds ratio, 1.08; 95% CI, 0.76–1.55; *p* = 0.66). Delaying surgery after cessation of antiplatelet drug did not affect risk of bleed recurrence. There were 15 in-hospital thromboembolic events recorded. Events were more common in the group pre-treated with antithrombotic drugs (3.3%) compared to the non-antithrombotic group (0.9%). Patients on an antithrombotic drug pre-operatively were at higher risk of thromboembolic events with no excess risk of bleed recurrence or worse functional outcome after CSDH drainage. The data did not support delaying surgery in patients on antithrombotic therapy. In the absence of a randomized controlled trial, early surgery and early antithrombotic recommencement should be considered in those at high risk of thromboembolic events.

## Introduction

Chronic subdural hematoma (CSDH) is a common neurosurgical condition in the older population. The use of antithrombotic drugs, either antiplatelets or anticoagulants, has increased in the past decade.^1^ Approximately 40% of patients with CSDH are taking an antithrombotic drug at the time of presentation.^2,3^ A large, population-based case-control study has demonstrated an association between increasing antithrombotic drug use and increasing incidence of CSDH.^4^ Perioperative management of antithrombotic drugs is therefore an important aspect for these patients.

Uncertainties remain over the perioperative management of antithrombotic therapy owing to limited robust data on this patient group. The therapeutic effect of commonly used vitamin K antagonists can be rapidly reversed. The same is not true of some direct oral anticoagulant drugs (DOACs) nor of antiplatelet medication. A recent national guideline provides recommendations for perioperative management of anticoagulation and -platelet therapy, but management requires assessment of individualized bleeding risks.^5^ The optimal timing of recommencing either anticoagulant or antiplatelet drugs is not known.^6^ Inconsistent conclusions were reached in several systematic reviews that evaluated hematoma recurrence risk in CSDH patients on antithrombotic drugs after surgical drainage.^2,7,8^ Importantly, few studies have investigated the risk of thromboembolic events in patients with CSDH.^2,8^ These uncertainties make clinical decision about antithrombotic drugs difficult.

We aimed to describe current UK practice in management of antithrombotic drugs perioperatively in patients with CSDH with the intent of informing future practice. We investigate the influence of antithrombotic drugs, including antiplatelets and -coagulants, on the risk of recurrence, thromboembolic events, and on unfavorable functional outcome.

## Methods

This report is reported according to the STROBE statement for cohort studies.^9^

### Study design and participants

This subsidiary study was based on a multi-center prospective cohort study conducted by the British Neurosurgical Trainee Research Collaborative. Study design has been described in the protocol^10^ and the primary report.^11^ In brief, 26 participating neurosurgical units identified and enrolled eligible participants between May 2013 and January 2014. Inclusion criteria were patients >16 years of age referred to a participating neurosurgical unit (NSU) with a CSDH confirmed on neuroimaging. We defined CSDH radiologically as a predominantly hypodense, isodense, or mixed-density subdural collection. We excluded patients with secondary CSDH attributed to underlying pathologies (e.g., subdural empyema, vascular malformations, and aneurysm). Details of antithrombotic medication and management of coagulation were available only in patients who were transferred to an NSU, so we restricted our analysis to this cohort. All participating NSUs received local clinical governance approval.

### Baseline and outcome variables

Data included demographics, medical comorbidities, history of traumatic brain injury (TBI) within 3 months before referral, pre-operative functional status as per the modified Rankin scale (mRS), pre-operative Glasgow Coma Score (GCS), management of pre-operative coagulation (use of platelet transfusion, vitamin K, fresh frozen plasma, and clotting factors), operative characteristics, pre-operative maximal thickness of CSDH, and post-operative bed rest. Decision to offer surgical management of CSDH was at the discretion of each NSU after clinical assessment.

Outcome variables included recurrence, functional outcome as mRS, thromboembolic events, and death. We defined recurrence as symptomatic and radiologically confirmed recurrent subdural collection requiring a repeat surgery within 60 days of index surgery. Discharge mRS score and persistent functional impairment were the functional outcomes. Persistent functional impairment is defined as same or lower mRS score at discharge compared with pre-operative mRS. Vaso-occlusive events included venous thromboembolism (deep vein thrombosis and pulmonary embolus), ischemic stroke, and myocardial infarction (type not specified) during admission. Inpatient deaths were recorded.

### Data sources

We identified eligible patients prospectively using local referral databases and operating theatre log books. Data collection of baseline and outcome variables was carried out prospectively. We submitted and stored the study data in a secure online database (Outcome Registry Intervention and Operation Network) that complies with UK government policies according to the Department of Health Information Governance and the Health and Social Care Information Centre.

### Bias and sample size

The multicenter prospective study, from which this substudy is derived, was designed with clear eligibility criteria, recruited from a representative population, and minimized selection bias. A pre-defined and published study protocol reduced misclassification of baseline and outcome variables. A comprehensive set of variables allowed for control of confounders. This aim of the primary study was to describe the contemporary management and outcomes of patients with CSDH, and therefore no sample size calculation was performed.

### Statistical analysis

We included patients with missing data in the analysis, when possible, and excluded them if the relevant data were missing for the particular analysis. The main comparison groups were patients using antithrombotic drugs on admission and patients without antithrombotic drugs. We conducted comparisons of baseline characteristics using chi-squared/Fisher's exact tests, where appropriate. Ordinal variables (e.g., mRS, GCS) were compared using non-parametric tests. Because of the non-Gaussian distribution of parametric data, the Mann-Whitney U test was used for comparisons in these variables. We used chi-squared test for univariate analysis of outcome variables. We conducted Cox regression and multiple logistic regression for multi-variate analysis for recurrence and functional outcome, respectively. Variables known to be associated with the outcome or found to be significantly different between the groups were entered into these multi-variate analyses. We restricted patients who underwent surgical management in the Cox regression analysis. Graphical hazard functions were generated from the Cox regression analyses. We performed paired analyses using the Wilcoxon rank-sum test on pre- and post-operative functional outcome. We used Stata software (version 13.0; StataCorp LP, College Station, TX) for all statistical analyses. A *p* value of <0.05 denoted statistical significance.

## Results

### Clinical characteristics

We included 817 patients with CSDH who were transferred to a neurosurgical unit and had data on pre-morbid antithrombotic drug use (Table 1). Three hundred fifty-three patients (43.2%) were on antithrombotic medication at the time of admission. The antithrombotic group were older, with a male predominance. Comorbidities relating to risk of thromboembolic events were more prevalent in the antithrombotic group (Table 1). The antithrombotic group had a lower pre-operative GCS and lower pre-operative functional status compared to the non-antithrombotic group.

**Table 1.** Baseline and Clinical Characteristics of 817 Patients with Chronic Subdural Hematoma

Characteristic	Antithrombotic group (*n* = 353)	Non-antithrombotic group (*n* = 464)	*p* value
Median age (IQR)	79 (73–84)	75 (63–84)	<0.01
Sex			0.05
Female	101 (28.6)	163 (35.1)	
Male	252 (71.4)	301 (64.9)	
Comorbidities			
Diabetes mellitus	70 (19.8)	63 (13.6)	0.02
Dementia	30 (8.5)	59 (12.7)	0.06
COPD	26 (7.4)	23 (5.0)	0.15
Cerebrovascular event	88 (24.9)	42 (9.1)	<0.01
Ischemic heart disease	133 (37.7)	73 (15.7)	<0.01
Arrhythmia	143 (40.5)	28 (6.0)	<0.01
Epilepsy	16 (4.5)	19 (4.1)	0.76
CSF shunt	1 (0.3)	6 (1.3)	0.12
Malignancy	34 (9.6)	35 (7.5)	0.29
Metallic heart valve	17 (4.8)	1 (0.2)	<0.01
Antithrombotic drugs			
Antiplatelet	171 (48.4)	—	
Vitamin K antagonist	148 (41.9)	—	
Other antithrombotics	9 (2.6)	—	
Dual antiplatelet & anticoagulant	11 (3.1)	—	
Dual antiplatelets	14 (4.0)	—	
History of TBI	216 (61.2)	294 (63.4)	0.53
Pre-operative mRS			0.02
mRS 0–3	191 (54.1)	290 (62.5)	
mRS 4–5	162 (45.9)	174 (37.5)	
Platelet transfusion	60 (17.0)	16 (3.5)	<0.01
Vitamin K	120 (34.0)	11 (2.4)	<0.01
Fresh frozen plasma	10 (2.8)	9 (1.9)	0.40
Clotting factors	99 (28.1)	2 (0.4)	<0.01
Pre-operative GCS			
Median (IQR)	14 (13–15)	14 (14–15)	<0.01
GCS 3–8	15 (4.3)	18 (3.9)	
GCS 9–12	49 (13.9)	54 (11.6)	
GCS 13–15	289 (81.9)	392 (84.5)	
Operation lateralisation			
Conservative	17 (4.8)	15 (3.2)	
Unilateral	257 (72.8)	343 (73.9)	
Bilateral	78 (22.1)	103 (22.2)	
Unknown	1 (0.3)	3 (0.7)	
Operation			<0.01
Burr hole drainage	276 (81.3)	410 (88.4)	
Minicraniotomy	37 (10.5)	33 (7.1)	
Others	11 (3.1)	3 (0.7)	
Conservative/unknown	18 (5.1)	18 (3.9)	
Drain inserted	279 (79.0)	374 (80.1)	0.69
Pre-operative maximal thickness	25 (18–31)	24 (17–30)	0.34
Post-operative bed rest			0.47
No restriction	142 (40.2)	175 (37.7)	
Instructed	193 (54.7)	271 (58.4)	
Unknown	18 (5.1)	18 (3.9)	

IQR, interquartile range; COPD, chronic obstructive pulmonary disease; CSF, cerebrospinal fluid; TBI, traumatic brain injury; mRS, modified Rankin scale; GCS, Glasgow Coma Score.

### Management of coagulation

In the antithrombotic group, the majority were taking either a single antiplatelet drug or a single anticoagulant drug (Table 1). In our cohort, no patients were taking DOACs. The most common drugs used were aspirin (41.4%), clopidogrel (6.8%), and warfarin (41.9%). Fourteen (4.0%) patients were on dual antiplatelet medications, and 11 (3.1%) were on dual antiplatelet and anticoagulant medications.

Discontinuation of antiplatelet drugs occurred at a median of 3 days before surgery (interquartile range [IQR], 1–7) and a median of 1 day before surgery (IQR, 1–4) in those taking anticoagulant drugs. The median day of discontinuation was shorter in patients on aspirin alone compared with clopidogrel alone (3 vs. 6 days; *p* = 0.02).

There were 58 (29.3%) adults using a single antiplatelet drug on admission who received pre-operative platelet transfusion. The most common anticoagulant reversal strategies were combination of vitamin K and clotting factors (52.7%), vitamin K alone (23.0%), and clotting factors alone (12.2%); the details of the clotting factors used were not collected in this survey. Reversal strategy was unavailable in 12.2% of adults on a pre-operative anticoagulant.

### Chronic subdural hematoma drainage management

Most patients (96.1%) underwent surgical management (Table 1). Burr-hole drainage was the commonest operation performed. The proportion of patients who underwent burr-hole drainage in the antithrombotic group was lower than that in the non-antithrombotic group (Table 1), but was similar between antiplatelet and -coagulant groups (Supplementary Table 1) (see online supplementary material at http://www.liebertpub.com). The use of drain and post-operation instructions regarding bed rest were similar in both groups.

### Post-operative recommencement of antithrombotics

Information was unavailable for 57 (28.8%) adults using pre-operative antiplatelet drug. In patients whose information was available, the time of antiplatelet recommencement was: 2 (1.4%) within 1 day, 8 (5.7%) between 1 and 2 days, 24 (17.0%) between 2 and 4 days, 34 (24.1%) after 4 days, and 73 (51.8%) to be reviewed by the general practitioner.

Information was unavailable for 23 (14.6%) adults using pre-operative anticoagulant drug. In patients whose information was available, the time of anticoagulant recommencement was: 6 (4.4%) within 1 day, 15 (11.1%) between 1 and 2 days, 21 (15.6%) between 2 and 4 days, 27 (20.0%) after 4 days, and 66 (48.9%) to be reviewed by the general practitioner.

### Recurrence

There were 795 patients with recurrence data available. Excluding the 31 conservatively managed patients with recurrence data, 76 (9.9%) of 764 patients suffered a recurrence within 60 days of their operation. The risk of recurrence was 10.1% and 9.9% in the antithrombotic and non-antithrombotic group, respectively (*p* = 0.93; Table 2). There was no significant difference in risk of recurrence between antiplatelet and anticoagulant groups (*p* = 0.15) or between aspirin and clopidogrel groups (*p* = 0.83). There was insufficient data available detailing the recommencement of antithrombotics to be able to make a comparison of rebleeding risk between those who restarted and those did not restart their antithrombotics within the time period examined.

**Table 2.** Summary Table of Outcome Measures among Surgically Treated Patients

	No. of events* n/*N (%)								
	Antithrombotic group	Non-antithrombotic group	*p* value	Antiplatelet	Warfarin	*p* value	Aspirin	Clopidogrel	*p* value
Recurrence	33/328 (10.1)	43/436 (9.9)	0.93	12/159 (7.6)	17/136 (12.5)	0.15	10/135 (7.4)	2/23 (8.7)	0.83
Discharge mRS 4–6	88/328 (26.8)	83/436 (19.0)	0.01	49/159 (30.8)	28/136 (20.6)	0.05	38/135 (28.2)	11/23 (47.8)	0.06
No functional improvement	122/328 (37.2)	152/436 (34.9)	0.59	66/159 (41.5)	45/136 (33.1)	0.14	54/135 (40.0)	12/23 (52.2)	0.27
Thromboembolic event	11/336 (3.3)	4/449 (0.9)	0.02	4/163 (2.5)	5/140 (3.6)	0.56	4/139 (2.9)	0/23 (0)	0.41

mRS, modified Rankin scale.

**FIG. 1.**
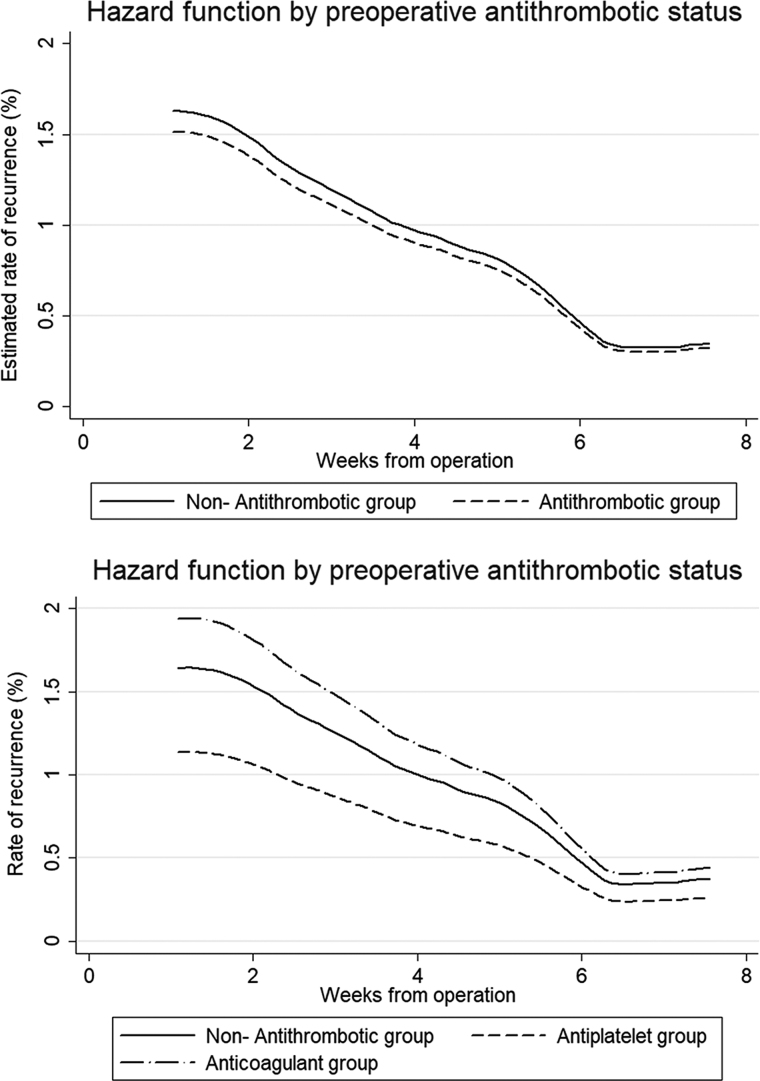
Hazard function for recurrence after operation using Cox regression. Graphs showing hazard for CSDH recurrence over time since index operation between non-antithrombotic and antithrombotic groups (above) and between non-antithrombotic group, antiplatelet group, and anticoagulant group (below).

The overall rate of crude recurrence was 1.2% per week (95% confidence interval [CI], 1.0–1.6) within 60 days of admission. The crude recurrence rate was 1.2% per week (95% CI, 0.9–1.6) in the non-antithrombotic group; 1.3% (95% CI, 0.9–1.8) in the antithrombotic group; 0.9% (95% CI, 0.5–1.6) in the antiplatelet group; and 1.6% (95% CI, 1.0–2.6) in the anticoagulant group. In the Cox regression analysis, the rate of recurrence in the antithrombotic group was similar to that in the non-antithrombotic group (hazard ratio, 0.93; 95% CI, 0.58–1.48; *p* = 0.76). Variables statistically associated with a higher rate of recurrence were male sex, lower pre-operative GCS, and no use of drain (Table 3). Rate of recurrence with time is shown in Figure 1. When stratifying antithrombotic groups, multivariate analysis did not show significant difference in recurrent rate between any treatment groups whereas the same variables remained associated with recurrence (Table 3).

**Table 3.** Multi-Variate Analysis Showing Hazard Ratios for Recurrence Using Cox Regression Model

	Hazard ratio	95% CI	*p* value
Model 1			
Antithrombotic drug use	0.93	0.58–1.48	0.76
Age (10-year interval)	1.13	0.93–1.39	0.22
Male sex	1.87	1.08–3.23	0.02
Preoperative GCS			
GCS 13–15	Ref	—	—
GCS 9–12	1.99	1.10–3.59	0.02
GCS 3–8	2.95	1.26–6.91	0.01
Drain insertion	0.38	0.23–0.62	<0.01
			
Model 2			
Antithrombotic drug use			
No antithrombotic drug use	Ref	—	—
Antiplatelet drug use	0.69	0.36-–.32	0.27
Anticoagulant drug use	1.18	0.67–2.10	0.57
Age (10-year interval)	1.13	0.92–1.38	0.25
Male sex	1.78	1.02–3.12	0.04
Preoperative GCS			
GCS 13–15	Ref	—	—
GCS 9–12	1.80	0.96–3.37	0.07
GCS 3–8	2.33	0.93–5.89	0.07
Drain insertion	0.43	0.25–0.72	<0.01

GCS, Glasgow Coma Score; CI, confidence interval.

Of 171 patients taking a single antiplatelet agent,167 had recurrence data available for analysis. The majority (142 patients; 85.5%) were taking aspirin. The overall median interval between discontinuing the antiplatelet drug and surgery was 3 days (IQR, 1–7 days). The median intervals for aspirin and clopidogrel groups were 3 (IQR, 1–7) days and 6 (IQR, 1.5–9.0) days, respectively. The antiplatelet group was divided into two groups on the basis of the median interval: those operated on before a 3-day washout and those operated on after at least 3 days. There were 6 (6.7%) recurrences in the 89 patients who had surgery within 3 days of stopping the antiplatelet drug; there were 6 (7.7%) recurrences in the 78 patients who had surgery after 3 days of discontinuing the antiplatelet drug. There was no statistically significant difference between these groups (*p* = 0.81). Restricting this analysis to the 142 patients on aspirin alone showed similar result (*p* = 0.89).

Data on timing of antiplatelet and anticoagulant medication recommencement were available in approximately half of the patients. A comparison of early (less than 4 days) or late (4 days or more) recommencement did not demonstrate any significant difference in the recurrence of symptomatic CSDH requiring surgery.

Of the 167 patients on a single antiplatelet agent, 49 (29.3%) patients received perioperative platelet transfusion. The median intervals between discontinuing the antiplatelet drug and the operation for patients with and without platelet transfusion were 1 and 5 days, respectively. There was no difference in the proportion of patients who received platelet transfusion between those on aspirin and those on clopidogrel (*p* = 0.55). The risk of recurrence was 8.2% in the platelet transfusion group and 6.8% in the non-transfusion group (*p* = 0.75).

There were 1 (7%) and 2 (18%) patients that suffered a recurrence in the dual antiplatelet and the dual antithrombotic group, respectively. Among the 31 patients managed conservatively, 3 suffered a symptomatic recurrence, of which 1 (6.3%) occurred in the antithrombotic group.

### Functional outcome

There were 171 (22.4%) patients who had an unfavorable functional outcome after CSDH surgery. Overall, the proportion of patients with unfavorable functional outcome (mRS 4–6) at discharge was higher in the antithrombotic group (Table 2). However, there was still an overall improvement in functional outcome in this antithrombotic group at discharge compared to pre-operative functional status (*p* < 0.01). This improvement was still observed in antiplatelet and anticoagulant groups analyzed separately and in non-antithrombotic groups (*p* < 0.01).

The unadjusted odd ratios (ORs) for persistent functional impairment at discharge are: 1.11 (95% CI, 0.82–1.49; *p* = 0.51) for antithrombotic group; 1.33 (95% CI, 0.91–1.93; *p* = 0.14) for antiplatelet group; and 0.92 (95% CI, 0.61–1.39; *p* = 0.70) for anticoagulant group. Adjusted ORs using the Mantel-Haenszel method are presented in Supplementary Table 2 (see online supplementary material at http://www.liebertpub.com).

In the multiple logistic regression, perioperative antithrombotic drug use was not associated with persistent functional impairment (OR, 1.08; 95% CI, 0.76–1.55; *p* = 0.66; Table 4). Separating antiplatelet and -coagulant groups in the analysis showed no association with functional improvement for pre-operative antiplatelet (OR, 1.05; 95% CI, 0.71–1.56; *p* = 0.81) and anticoagulant (OR, 1.30; 95% CI, 0.60–2.83; *p* = 0.51) drug use.

**Table 4.** Multi-Variate Analysis Showing Odds Ratios for Persistent Functional Impairment Using Logistic Regression

	Odds ratio	95% CI	*p* value
Model 1			
Antithrombotic drug use	1.08	0.76–1.55	0.66
Age (10-year interval)	1.38	1.20–1.58	<0.01
History of stroke	1.38	0.91–2.09	0.13
Vitamin K	1.26	0.61–1.87	0.83
Clotting factors	0.47	0.25–0.89	0.02
Preoperative GCS			
GCS 13–15	Ref	—	—
GCS 9–12	1.15	0.74–1.80	0.54
GCS 3–8	1.07	0.49–2.32	0.87
Drain insertion	0.79	0.52–1.18	0.25
			
Model 2			
Antithrombotic drug use			
No antithrombotic drug use	Ref	—	—
Antiplatelet drug use	1.05	0.71–1.56	0.81
Anticoagulant drug use	1.30	0.60–2.83	0.51
Age (10-year interval)	1.38	1.20–1.58	<0.01
History of stroke	1.36	0.89–2.09	0.16
Vitamin K	0.92	0.44–1.93	0.84
Clotting factors	0.49	0.23–1.01	0.05
Preoperative GCS			
GCS 13–15	Ref	—	—
GCS 9–12	1.17	0.73–1.87	0.65
GCS 3–8	1.13	0.52–2.49	0.76
Drain insertion	0.72	0.48–1.10	0.13

GCS, Glasgow Coma Score; CI, confidence interval.

There were 30 conservatively managed patients with functional outcome available. Most of them (80%) had a favorable functional outcome on discharge. Compared to their admission functional status, 40% remained the same, 43.3% improved, and 16.7% worsened in their functional status.

### Vaso-occlusive events

Data were available in 817 patients. There were 15 vaso-occlusive events reported: 11 events in the antithrombotic group (Table 2). The antithrombotic group had a higher rate of vaso-occlusive events compared to non-antithrombotic group (OR, 3.70; 95% CI, 1.16–11.80; *p* = 0.02; Table 2). Compared to the non-antithrombotic group, the anticoagulant group (OR, 4.02; 95% CI, 1.06–15.30; *p* = 0.03), but not antiplatelet group (OR, 2.75; 95% CI, 0.68–11.2; *p* = 0.14), also had a higher rate of vaso-occlusive events.

## Discussion

There are significant variations in perioperative management of antithrombotics in patients with CSDH treated in the UK and Ireland. This may reflect an individualized approach to risk assessment, but may also result from the lack of robust evidence to guide practice. An approach favoring early surgery and early post-operative recommencement on antithrombotics may reduce the risk of vaso-occlusive complications without increasing the risk of CSDH recurrence.

Previous systematic reviews^7,8^ and a meta-analysis^2^ have yielded inconsistent conclusions regarding the risk of CSDH recurrence after an operation in patients on an antithrombotic medication at CSDH diagnosis. These inconsistencies may stem from the differing study design of CSDH studies.^2,7,8,12^ In our secondary analysis of this large cohort, we found no discernible difference in recurrence risk among those on previous antithrombotic drugs. A longer washout period of antiplatelet before an operation did not provide observable benefits. This may be because the full recovery of platelet function takes around 4 days and 10 days after cessation of aspirin and clopidogrel, respectively,^13^ which is longer than the interval to surgery in most patients in this study. Our data indicate that modest increase in the washout period does not confer an advantage in terms of reducing post-operative bleed recurrence. In the absence of strong evidence for delayed surgery for patients on antiplatelet drugs, early surgery may be appropriate to minimize time in hospital and time without antiplatelet drugs.

Platelet transfusion may aid in preventing or reducing bleeding from subdural veins during surgery that later presents as recurrent CSDH, although the actual mechanism of CSDH recurrence after surgery is not known. We found no compelling benefit for the use of platelet transfusion in patients on pre-operative antiplatelet medications. Although there is no clinical trial examining the effect of platelet transfusion in CSDH patients, a recent randomized trial on the use of platelet transfusion in spontaneous cerebral hemorrhage associated with antiplatelet therapy (PATCH) found worse functional outcome and serious adverse effects in patients receiving platelet transfusion.^14^ This finding may not be directly applicable to CSDH patients given that the pathophysiology of the two conditions are different. In our data, patients receiving a platelet transfusion had a shorter time between stopping antiplatelet drug and surgery. The longer time to surgery in the no-platelet group may have had the effect of allowing native platelet function to further recover, explaining the lack of observed benefit of a platelet transfusion in terms of symptomatic CSDH recurrence. Evidence for the use of platelet transfusion needs to be clarified by understanding the pathological processes in CSDH as well as further large observational studies.

Dual antiplatelet therapy and dual antithrombotic therapy carry a higher risk of bleeding. A large Danish national cohort study investigating the risk of bleeding in dual antithrombotic therapy suggests an increased risk of intracranial bleeding in this group.^15^ The incidence of subdural hematoma is higher in patients on dual antiplatelet drugs compared to aspirin alone in a systematic review of randomized trials.^16^ Our results suggest a similar risk of recurrence with pre-operative dual antiplatelet therapy and a higher risk of recurrence with concurrent antiplatelet and -coagulant use. But this subgroup on dual therapy is underpowered. Given that this group is also at higher risk of vaso-occlusive events, further clarification would address the balance of risks.

Current clinical guidelines on perioperative antithrombotic use emphasize an individual risk assessment with no clear guidance on the optimal timing of recommencing antithrombotic drugs.^5^ An international survey of perioperative acetylsalicylic acid use in CSDH patients^6^ confirms the significant variation in clinical practice shown in previous systematic reviews^2,8^ and in our analysis. One retrospective cohort study compared outcomes in those who restarted on their antithrombotic drugs to those who did not.^3^ The researchers found similar risks of thromboembolism (unadjusted OR, 1.34; 95% CI, 0.29–6.13), but a lower risk of recurrence (unadjusted OR, 0.06; 95% CI, 0.02–0.20) in the antithrombotic restart group. This result, however, is limited by few outcome events in the study group and likely confounders that were not adjusted for. A recent retrospective study compared outcomes between patients restarted on antithrombotic drugs within 30 days and those restarted after 30 days.^17^ They reported recurrence risks of 7% versus 14% (*p* = 0.08) and risk of thromboembolic events of 2% versus 11% (*p* = 0.01) for early and late resumption groups, respectively. Most of the thromboembolic events (63%) occurred within 1 month of CSDH surgery. It is difficult to apply these findings because of the lack of analyses adjusting for known confounders and possible reporting bias attributed to unspecified methods of obtaining thromboembolic outcomes. However, from these observations together with our own findings, the suggestion emerges that patients on previous antithrombotic drugs are at a higher risk of thromboembolic events, but similar risk of recurrence compared to patients not on antithrombotic drugs. The risks of these complications appear to be highest within the first month after surgery. Future studies that focus on understanding particular characteristics of this high-risk group that influence the balance of risks, perhaps utilizing existing risk scores such as CHA_2_DS_2_-VASc^18^ and HAS-BLED,^19^ would help in systematic and individualized risk assessment.

Another consideration is the prescription of chemical venous thromboembolism (VTE) prophylaxis post-operatively. Patients with CSDH are at risk of VTE events given that they are often of an older age and have prolonged immobility. Chemical VTE prophylaxis protocols vary between neurosurgical centres.^20–22^ One retrospective study with uniform chemical VTE prophylaxis strategy showed that giving VTE prophylaxis on post-operative day 1 was associated with a higher risk of recurrence compared to no chemical prophylaxis (32% vs. 19%).^23^ There was no VTE event observed during the in-hospital follow-up period. Other studies with variable practice reported no increased risk of recurrence associated with VTE prophylaxis use.^24,25^ Compression stockings, and intermittent pneumatic compression in those bed-bound, may be the safer options for VTE prophylaxis, especially in those on previous antithrombotic drugs. As recommended by the available guideline,^5^ an individualized risk assessment should dictate the most appropriate management.

We observed 77.6% patients who had a favorable functional outcome at discharge, which is similar to the 75.5% reported in a clinical trial.^26^ Our findings also showed that patients on previous antithrombotic drug had a worse functional outcome at discharge. This is most likely because of their poorer admission functional status given that this association has previously been described.^11^ Future studies should address the longer-term functional outcome in patients on an antithrombotic drug in conjunction with recurrence and vaso-occlusive risks. The intermediate and longer-term risk profile of those on an antithrombotic drug may diverge from that of those on no antithrombotic drugs. The risk profile of recurrence and risk of thromboembolic events may help future studies to be sufficiently powered to detect clinically significant differences in outcomes.

In presenting these data, uncontrolled selection bias and unmeasured confounders may be present given that there were no pre-defined criteria for transferring a patient to an NSU. However, the multi-center study design and large sample size helped to reduce the effects of these limitations. The smaller subgroup cohorts according to their antithrombotic agent preclude multi-variate analyses because of the few outcomes occurring. We were unable to investigate the effect of antithrombotics on outcomes in patients who were managed conservatively outside the NSUs. Information on the indication for antithrombotics use was unavailable, limiting the description of our cohort. Strategies for post-operative venous thromboembolism prophylaxis (mechanical ± chemical prophylaxis) were not collected, which would have an impact on the number of vaso-occlusive events.

Our study only measured short-term recurrence within 60 days, which is a shorter period than most studies in the literature reporting the risk of recurrence in antithrombotics groups.^2^ The subsequent smaller number of outcomes may reduce our ability to observe any difference between comparison groups. But the effect of this is likely to be small given the plateauing of recurrence rate after 6 weeks. Only vaso-occlusive events that occurred during hospital admission were recorded. Given that vaso-occlusive events were not a primary outcome and reporting depended on the local investigator, our study may underestimate the risk of vaso-occlusive events. We were unable to examine the post-operative management of antithrombotic and vaso-occlusive events because of insufficient information. With the increasing prescription of DOACs and decline in warfarin use,^27^ our findings may not apply to patients on DOACs. Guidance on the management of DOACs perioperatively is available.^5^ Future studies can specifically address this aspect of clinical practice, which would reflect more on the changing contemporary patient cohort.

## Conclusion

Pre-operative antithrombotic use is not associated with worse clinical outcomes after CSDH surgery. However, patients on an antithrombotic drug before presentation, which is stopped for surgery, are at subsequent higher risk of vaso-occlusive events. There is no clear benefit for delaying surgery in patients on antiplatelet drugs. Early surgery and antithrombotic drug recommencement can minimize morbidity associated with longer hospital stay and period without antithrombotic drugs.

To determine the optimal time of antithrombotic resumption, both the absolute risks and the impact of the complication, for example, reoperation, extracranial bleeding, and functional impairment, need to also be further investigated and this may be possible as part of a well-constructed prospective randomized controlled trial.

## British Neurosurgical Trainee Research Collaborative (BNTRC)

### Collaborating authors

The following acted as either local trainee or consultant principal investigators: Afshari FT, Ahmed AI, Alli S, Al-Mahfoudh R, Bal J, Belli A, Borg A, Bulters D, Carleton-Bland N, Chari A, Coope D, Coulter IC, Cowie CJ, Critchley G, Dambatta S, D'Aquino D, Dhamija B, Dobson G, Fam MD, Gray WP, Grover PJ, Halliday J, Hamdan A, Hill CS, Hutchinson PJ, Jamjoom AAB, Joannides AJ, Jones TL, Joshi SM, Kailaya-Vasan A, Karavasili V, Khan SA, King AT, Kuenzel A, Livermore LJ, Lo W, Marcus HJ, Martin J, Matloob S, Mitchell P, Mowle D, Narayanamurthy H, Nelson RJ, Ngoga D, Noorani I, O'Reilly G, Othman H, Owusu-Agyemang K, Manjunath Prasad KS, Plaha P, Pollock J, Prasad KS, Price R, Pringle C, Ray A, Reaper J, Scotton W, Shapey J, Simms N, Smith S, Statham P, Steele L, St George J, Stovell MG, Tarnaris A, Teo M, Thomson S, Thorne L, Vintu M, Whitfield P, Wilson M, Wilby M, Woodfield J, and Zaben M.

## Supplementary Material

Supplemental data
