# Supplementary material for: Influence of Antiplatelet and Anticoagulant Drug Use on Outcomes after Chronic Subdural Hematoma Drainage
Source: J Neurotrauma. 2021 Apr 5;38(8):1177–84. doi: 10.1089/neu.2018.6080 (PMC8060161; doi:10.1089/neu.2018.6080)
Supplement: Supplemental data [file Supp_Data.pdf]

Supplementary Data

STROBE Statement: Checklist of Items That Should Be Included in Reports of Cohort Studies

	Item no.	Recommendation	Page
Title and abstract	1	(*a*) Indicate the study's design with a commonly used term in the title or the abstract.	2
		(*b*) Provide in the abstract an informative and balanced summary of what was done and what was found.	4
Introduction			
Background/rationale	2	Explain the scientific background and rationale for the investigation being reported.	5
Objectives	3	State specific objectives, including any pre-specified hypotheses.	5
Methods			
Study design	4	Present key elements of study design early in the paper.	5
Setting	5	Describe the setting, locations, and relevant dates, including periods of recruitment, exposure, follow-up, and data collection.	5
Participants	6	(*a*) Give the eligibility criteria, and the sources and methods of selection of participants. Describe methods of follow-up.	5–6
		(*b*) For matched studies, give matching criteria and number of exposed and unexposed.	n/a
Variables	7	Clearly define all outcomes, exposures, predictors, potential confounders, and effect modifiers. Give diagnostic criteria, if applicable.	6
Data sources/ measurement	8[a]	For each variable of interest, give sources of data and details of methods of assessment (measurement). Describe comparability of assessment methods if there is more than one group.	6
Bias	9	Describe any efforts to address potential sources of bias.	6
Study size	10	Explain how the study size was arrived at.	6–7
Quantitative variables	11	Explain how quantitative variables were handled in the analyses. If applicable, describe which groupings were chosen and why.	7
Statistical methods	12	(*a*) Describe all statistical methods, including those used to control for confounding.	7
		(*b*) Describe any methods used to examine subgroups and interactions.	7
		(*c*) Explain how missing data were addressed.	7
		(*d*) If applicable, explain how loss to follow-up was addressed.	7
		(*e*) Describe any sensitivity analyses.	7
Results			
Participants	13[a]	(a) Report numbers of individuals at each stage of study—for example, numbers potentially eligible, examined for eligibility, confirmed eligible, included in the study, completing follow-up, and analyzed.	7
		(b) Give reasons for non-participation at each stage.	7
		(c) Consider use of a flow diagram.	n/a
Descriptive data	14[a]	(a) Give characteristics of study participants (e.g., demographic, clinical, or social) and information on exposures and potential confounders.	7
		(b) Indicate number of participants with missing data for each variable of interest.	7–17
		(c) Summarize follow-up time (e.g., average and total amount).	7
Outcome data	15[a]	Report numbers of outcome events or summary measures over time	Table 2
Main results	16	(*a*) Give unadjusted estimates and, if applicable, confounder-adjusted estimates and their precision (e.g., 95% confidence interval). Make clear which confounders were adjusted for and why they were included.	9–17
		(*b*) Report category boundaries when continuous variables were categorized.	n/a
		(*c*) If relevant, consider translating estimates of relative risk into absolute risk for a meaningful time period.	n/a
Other analyses	17	Report other analyses done—for example, analyses of subgroups and interactions, and sensitivity analyses.	9–17
Discussion			
Key results	18	Summarize key results with reference to study objectives.	17–19
Limitations	19	Discuss limitations of the study, taking into account sources of potential bias or imprecision. Discuss both direction and magnitude of any potential bias.	19
Interpretation	20	Give a cautious overall interpretation of results considering objectives, limitations, multiplicity of analyses, results from similar studies, and other relevant evidence.	20
Generalizability	21	Discuss the generalizability (external validity) of the study results.	19–20
Other information			
Funding	22	Give the source of funding and the role of the funders for the present study and, if applicable, for the original study on which the present article is based.	20

Note: An Explanation and Elaboration article discusses each checklist item and gives methodological background and published examples of transparent reporting. The STROBE checklist is best used in conjunction with this article (freely available on the Web sites of PLoS Medicine at http://www.plosmedicine.org/, Annals of Internal Medicine at http://www.annals.org/, and Epidemiology at http://www.epidem.com/). Information on the STROBE Initiative is available at http://www.strobe-statement.org.

[a]Give information separately for exposed and unexposed groups.

Supplementary Table S1. Baseline and Clinical Characteristics of Patients on Pre-Operative Antiplatelet or Anticoagulant Drug with Chronic Subdural Hematoma Compared to Non-Antithrombotic Group

Characteristic	*Antiplatelet only group* (n = *171)*	*Anticoagulant only group* (n = *148)*	*Non-antithrombotic group* (n = *464)*
Median age (IQR)	79 (73–84)	79 (71.0–84.5)	75 (63–84)
Sex			
Female	47 (27.5)	41 (27.7)	163 (35.1)
Male	124 (72.5)	107 (72.3)	301 (64.9)
Comorbidities			
Diabetes mellitus	38 (22.2)	29 (19.6)	63 (13.6)
Dementia	18 (10.5)	11 (7.4)	59 (12.7)
COPD	12 (7.0)	11 (7.4)	23 (5.0)
Cerebrovascular event	51 (29.8)	29 (19.6)	42 (9.1)
Ischemic heart disease	68 (39.7)	46 (31.1)	73 (15.7)
Arrhythmia	24 (14.0)	106 (71.6)	28 (6.0)
Epilepsy	9 (5.3)	6 (4.1)	19 (4.1)
CSF shunt	1 (0.6)	0 (0)	6 (1.3)
Malignancy	17 (9.9)	15 (10.1)	35 (7.5)
Metallic heart valve	1 (0.6)	14 (9.5)	1 (0.2)
History of TBI	112 (65.5)	81 (54.7)	294 (63.4)
Pre-operative mRS			
mRS 0–3	86 (50.3)	90 (60.8)	290 (62.5)
mRS 4–5	85 (49.7)	58 (39.2)	174 (37.5)
Platelet transfusion	49 (28.7)	2 (1.4)	16 (3.5)
Vitamin K	1 (0.6)	112 (75.7)	11 (2.4)
Clotting factors	0 (0)	7 (4.8)	2 (0.4)
Pre-operative GCS			
Median (IQR)	14 (13–15)	14 (14–15)	14 (14–15)
GCS 3–8	6 (3.5)	8 (5.4)	18 (3.9)
GCS 9–12	25 (14.6)	14 (9.5)	54 (11.6)
GCS 13–15	140 (81.9)	126 (85.1)	392 (84.5)
Operation lateralisation			
Conservative	8 (4.7)	8 (5.4)	15 (3.2)
Unilateral	131 (76.6)	102 (68.9)	343 (73.9)
Bilateral	31 (18.1)	38 (25.7)	103 (22.2)
Unknown	1 (0.6)	0 (0)	3 (0.7)
Operation			
Burr hole drainage	138 (80.7)	120 (81.1)	410 (88.4)
Minicraniotomy	18 (10.5)	16 (10.8)	33 (7.1)
Others	6 (3.5)	4 (2.7)	3 (0.7)
Conservative/unknown	9 (5.3)	8 (5.4)	18 (3.9)
Drain inserted	135 (79.0)	118 (79.7)	374 (80.1)
Pre-operative maximal thickness	25 (19–31)	24 (17–30)	24 (17–30)
Post-operative bed rest			
No restriction	69 (40.4)	54 (36.5)	175 (37.7)
Instructed	93 (54.4)	86 (58.1)	271 (58.4)
Unknown	9 (5.3)	8 (5.4)	18 (3.9)

IQR, interquartile range; COPD, chronic obstructive pulmonary disorder; CSF, cerebrospinal fluid; mRS, TBI, traumatic brain injury; modified Rankin scale; GCS, Glasgow Coma Score

Supplementary Table S2. Crude and Adjusted OR of Antithrombotic Drug Use for Persistent Functional Impairment at Discharge

	Any antithrombotic drugs		*Antiplatelet drugs*		*Anticoagulant drugs*	
	OR (95% CI)	p *value*	*OR (95% CI)*	p *value*	*OR (95% CI)*	p *value*
Crude	1.11 (0.82–1.49)	0.51	1.33 (0.91–1.93)	0.14	0.92 (0.61–1.39)	0.70
Adjusted for						
Agegroup	0.95 (0.69–1.30)	0.74	1.13 (0.76–1.67)	0.55	0.83 (0.54–1.27)	0.38
Sex	1.12 (0.83–1.50)	0.47	1.32 (0.91–1.91)	0.14	0.93 (0.62–1.39)	0.72
Diabetes	1.10 (0.82–1.49)	0.52	1.30 (0.89–1.89)	0.17	0.93 (0.62–1.40)	0.73
Cerebrovascular disease	1.02 (0.75–1.39)	0.91	1.16 (0.78–1.72)	0.46	0.90 (0.60–1.36)	0.62
Ischemic heart disease	1.08 (0.80–1.47)	0.62	1.27 (0.87–1.86)	0.21	0.89 (0.59–1.34)	0.57
Arrhythmia	1.19 (0.86–1.64	0.30	1.33 (0.91–1.93)	0.14	0.90 (0.53–1.55)	0.71
Metallic heart valve	1.11 (0.82–1.50)	0.50	1.34 (0.92–1.94)	0.13	0.91 (0.60–1.40)	0.67
Preoperative GCS	1.10 (0.82–1.48)	0.53	1.31 (0.90–1.91)	0.15	0.93 (0.62–1.39)	0.71
Drain insertion	1.10 (0.81–1.48)	0.54	1.30 (0.90–1.90)	0.16	0.92 (0.62–1.39)	0.71
Platelet transfusion	1.10 (0.81–1.49)	0.80	1.34 (0.90–2.02)	0.15	0.93 (0.62–1.40)	0.73
Vitamin K administration	1.25 (0.90–1.74)	0.18	1.32 (0.91–1.92)	0.14	1.15 (0.59–2.24)	0.69
Fresh frozen plasma	1.09 (0.81–1.48)	0.55	1.36 (0.94–1.98)	0.10	0.89 (0.59–1.34)	0.58
Clotting factors	1.37 (0.99–1.89)	0.06	1.32 (0.91–1.91)	0.15	1.52 (0.84–2.74)	0.17
Postoperative bed rest	1.12 (0.83–1.52)	0.45	1.34 (0.92–1.95)	0.13	0.92 (0.61–1.40)	0.70

OR, odds ratio; GCS, Glasgow Coma Score; CI, confidence interval.
